# *TP53*/p53 alterations and Aurora A expression in progressor and non-progressor colectomies from patients with longstanding ulcerative colitis

**DOI:** 10.3892/ijmm.2014.1974

**Published:** 2014-10-20

**Authors:** MARIANN FRIIS-OTTESSEN, ESPEN BURUM-AUENSEN, AASA R. SCHJØLBERG, PER OLAF EKSTRØM, SOLVEIG N. ANDERSEN, OLE PETTER CLAUSEN, PAULA M. DE ANGELIS

**Affiliations:** 1 Division of Diagnostics and Intervention, Department of Pathology, Oslo University Hospital, Rikshospitalet, Oslo, Norway; 2 Medivir Norge, University of Oslo, Oslo, Norway; 3 Department of Pathology, University of Oslo, Oslo, Norway; 4 Division of Surgery and Cancer Medicine, Institute for Cancer Research, The Norwegian Radium Hospital, Oslo University Hospital, Oslo, Norway; 5 Department of Pathology, Akershus University Hospital, Division of Medicine and Laboratory Sciences, University of Oslo, Oslo, Norway

**Keywords:** ulcerative colitis, aneuploidy, dysplasia, immunohistochemistry, p53, Aurora

## Abstract

Aneuploidy is a common feature in the colonic mucosa of patients suffering from the inflammatory bowel disease ulcerative colitis (UC) and often precedes the development of dysplasia and cancer. Aneuploidy is assumed to be caused by missegregation of chromosomes during mitosis, often due to a faulty spindle assembly checkpoint. p53 is a tumour suppressor protein known to regulate the spindle assembly checkpoint and is frequently mutated in aneuploid cells. Aurora A is a presumed oncoprotein, also involved in regulation of the spindle assembly checkpoint. In the present study, we examined the mutational frequency of *TP53* and the protein levels of p53 in a set of 20 progressor and 10 non-progressor colectomies from patients suffering from longstanding UC. In addition, we re-examined previously published immunohistochemical data on Aurora A expression using the same material. Levels of Aurora A were re-examined with regard to DNA ploidy status and dysplasia within the progressors, as well as in relation to p53 accumulation and *TP53* mutational status. We detected p53 accumulation only within the progressor colectomies, where it could be followed back 14 years prior to the colectomies, in pre-colectomy biopsies. *TP53* mutations were detected in both progressors and non-progressors. Expression levels of Aurora A were similar in the progressors and non-progressors. Within the group of progressors however, low levels of Aurora A were associated with areas of DNA aneuploidy, as well as with increasing degrees of dysplasia. Our results indicate that alterations in p53 may be an early biomarker of a progressor colon, and that p53 is accumulated early in UC-related carcinogenesis. Furthermore, a decreased Aurora A expression is associated with the development of DNA aneuploidy, as well as with dysplasia in UC progressors.

## Introduction

Ulcerative colitis (UC) is an inflammatory bowel disease that predisposes for colorectal cancer. The risk of developing malignancies increases with disease duration and also with age at disease onset (1). Malignant development in UC is a multistep progression through inflammation, regeneration and dysplasia, leading to adenocarcinoma. The process also includes a number of important molecular changes. The colonic mucosa of patients with UC may harbour severe molecular abnormalities due to chromosomal instability (CIN), leading to DNA aneuploidy. Aneuploidy is regarded as an early event in the malignant development of UC (2) and may be present in both dysplastic, as well as in non-dysplastic colonic mucosa (3,4).

Aneuploidy in UC relates to disease duration (5–8) and is regarded as an independent risk factor for the development of adenocarcinoma in UC (9,10). The consequence of aneuploidy in non-neoplastic cells is growth arrest or cell death, but is in UC suggested to be a precursor of future malignancies (6). More than half of the colorectal adenocarcinomas developing from UC present DNA aneuploidy (7,9), and it is thus considered a major contributor to the neoplastic phenotype (11,12).

The term aneuploidy refers to either structural errors or copy number errors in chromosomes, and aneuploid cells usually contain a combination of these two errors. Structural errors are most likely products of chromosomal breakage, and, whereas multiple mechanisms may underlie chromosomal breakage (13–15), copy number errors are achieved mainly through errors in chromosomal segregation (16,17). The spindle checkpoint (or mitotic checkpoint) is crucial for the separation of chromosomes to both daughter cells during mitosis. It is a complex signalling cascade that will arrest mitosis upon faulty alignment of chromosomes or if the spindle fails to attach to kinetochores properly (18–20). A dysfunctional spindle checkpoint is considered to be a major cause of aneuploidy in malignancies (16,17,21).

p53 is a tumour suppressor protein with multiple functions in the regulation of the cell cycle and chromosomal stabilization (22). In cancers, there are often mutations and/or loss of heterozygosity in the *TP53* gene, resulting in loss of function. In UC-related carcinogenesis, evidence points to the inactivation of p53 as being a relatively early event (22–24), whereas it is considered a late event in the development of sporadic colorectal cancers (25). p53 and Aurora A are reportedly involved in a mitotic feedback loop: p53 is considered to be a negative regulator of Aurora A expression, whereas Aurora A can phosphorylate p53 rendering it incapable of binding to DNA, or marking it for degradation (22,26–28). If wild-type p53 is assumed to be a negative regulator of the mitotic spindle kinase Aurora A (22,29), the loss of functional p53 may have serious implications for regulation of the spindle checkpoint. Loss of wild-type p53 function may result in centrosome amplification, faulty chromosomal segregation and aneuploidy. In the absence of *TP53* mutations, the accumulation of p53 in a UC colon can also be due to a programmed p53 response to various reactive oxidative species present in inflamed tissue (30).

Overexpression of Aurora A is implicated in abnormal centrosome amplification and in the abrogation of the spindle checkpoint (31). The gene coding for Aurora A is located on 20q13.2, a chromosomal arm frequently amplified in solid tumours, including colorectal tumours (32). The expression of Aurora A has been reported to be elevated in several tumour types (33,34), as well as in the colonic mucosa of patients with UC (35).

In this study, we have assessed both the mutational frequency of *TP53* and the protein levels of p53 in a set of colectomies from patients suffering from longstanding UC. We also re-evaluated previously published data on Aurora A expression assessed by immunohistochemical staining in the same colectomies (35). The colectomies were stratified as progressors and non-progressors, as previously presented (36,37). Within the progressors, we assessed the results from Aurora A in association with DNA ploidy status and advancing degrees of dysplasia, as well as with the protein levels of p53 and the *TP53* mutation status.

## Materials and methods

### UC colectomies and patients

Thirty patients suffering from longstanding UC were included in this study. All patients had suffered from UC for >10 years prior to the colectomy, some as long as 30 years. Patients also varied widely with respect to age at the time of the first presentation of symptoms (from 10 to 60 years old).

The colectomy specimens have been previously described (35–37). We divided the colectomies into progressors and non-progressors, revealing 10 non-progressors that did not present any dysplastic lesions or DNA aneuploidy, and 20 progressors that all presented at least one area of dysplasia/cancer, where the majority of cases also presented lesions with DNA aneuploidy.

At least 8 locations from each colectomy were examined, and within the progressors, we found 83 non-dysplastic areas, 31 areas indefinite for dysplasia, 29 areas with dysplasia and 8 adenocarcinomas. A total of 18 non-dysplastic and 20 dysplastic areas revealed DNA aneuploidy. The aneuploid, dysplastic areas included 8 areas of indefinite dysplasia and 5 adenocarcinomas. By definition, all non-progressor lesions were diploid and non-dysplastic. Detailed distributions of dysplasia and aneuploid lesions within the progressors have been previously described (35,37).

### Ethical considerations

The use of this material for research purposes has ethical approval from the Regional Ethics Committee (approval no. REK S-06062).

### Tissue microarray evaluation

Tissue microarrays (TMAs) from 8 locations within each colon specimen were prepared using a Beecher tissue microarray instrument (Beecher Instruments, Inc., Sun Prairie, WI, USA) as previously described (35). The core size was 0.6 mm. All cores had been previously evaluated by an experienced pathologist (O.P.C.). At least 2 tissue cores from each mucosal region were sampled.

TMAs do not consistently display full colonic crypts as whole sections do. p53 staining was performed on whole sections since the detectable accumulation of p53 is heterogeneous (positively- and negatively-stained areas in the same section). If tissue cores for TMAs were sampled from areas negative for p53, this may have led to an increased number of false negatives. The expression of Aurora A was homogeneous (staining was evenly distributed throughout the section); thus, TMAs were regarded as reliable for the estimation of Aurora A protein expression.

### Immunohistochemistry (IHC)

Immunohistochemical staining for p53 and Aurora A was performed as described in our previous publications (35,38,39).

p53 accumulation was assessed microscopically, by manually counting positive nuclei in whole sections, as previously described (38). At least 1,200 nuclei were counted, and a section was scored as positive for p53 if >5% of the cells in a section showed nuclear staining as previously presented by our research group (40).

**Figure 1:**
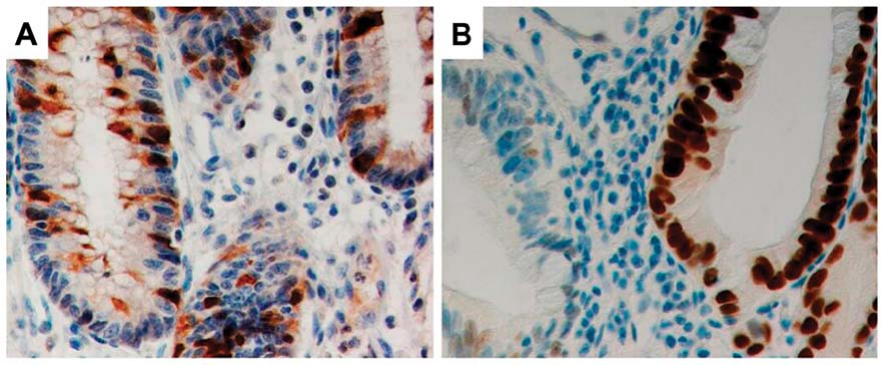
Immunohistochemical staining of (A) Aurora A and (B) p53 in dysplastic ulcerative colitis (UC) colonic mucosa. Aurora A staining of non-dysplastic UC mucosa and non-UC control has been previously presented (35). p53 staining illustrates heterogeneity, showing one positive and one negative crypt. Images are shown at ×400 magnification.

Aurora A expression was assessed from TMAs. Aurora A protein expression was defined for each sample as the percentage of positive cells out of at least 300 randomly selected mucosal epithelial cells from each included tissue core. Typical staining of Aurora A and p53 is presented in Fig. 1. With increasing degree of dysplasia, an increasing amount of cells with nucleic positivity of Aurora A also presented cytoplasmic staining.

### p53 mutation analysis

Mutation analysis for *TP53* exons 5–8 was performed by cycling temperature capillary electrophoresis (CTCE), as previously described (41,42). This procedure detects the presence of mutations. We did not sequence the mutation-positive cases in order to determine the actual mutation. The primer sequences for the mutation analyses are presented in the study by Bjørheim *et al* (43).

### Statistical analysis

p53/*TP53* correlations were examined in cross tabulation and assessed by Pearson’s χ^2^ test. Assessment of Aurora A protein levels in association with the DNA ploidy status, mucosal morphology and p53 accumulation, as well as the *TP53* mutational status, was performed using a multilevel model compensating for patient differences, as each patient included in this study contributed with more than one biopsy. A linear mixed model (LMM) with restricted maximum likelihood (REML) estimations and a Bonferroni post hoc test were used. Tests were performed with PASW statistics 18 (Chicago, IL, USA). All tests were two-sided and a p-value of 0.05 was considered to indicate a statistically significant difference.

## Results

### p53 immunohistochemistry

No accumulation of p53 was detected within the 10 non-progressor colectomies. Of the 20 progressor colectomies, 60% (12/20) harboured areas with accumulation of p53, but no colectomy specimens showed p53 accumulation through all 8 lesions. The 20 progressors had a total of 130 lesions available for p53 assessment, and 20.8% (27/130) of the lesions were positive for p53 accumulation. Within the positive lesions, 22.2% (6/27) also contained aneuploid populations. In precolectomy biopsies from patients with colectomies positive for p53 accumulation, we detected p53 accumulation up to 14 years prior to colectomy. A summary of colectomy lesions positive for p53 accumulation, including DNA ploidy status and mucosal morphology, is presented in Table I.

### Mutation analysis of the TP53 gene (exons 5–8)

TP53 mutations were found in both progressors and non-progressors. A total of 70% (7/10) of the non-progressors and 55% (11/20) of the progressors harboured areas with mutations in one of the *TP53* mutation hotspots (exons 5–8).

Of the 70 non-progressor lesions available for *TP53* mutation analysis 20% (14/70) had a *TP53* mutation. The 20 progressor colectomies yielded 129 lesions available for *TP53* mutation analysis. Of these 129 lesions, 11.6% (15/129), harboured a mutation in one of the mutation hotspots examined (exons 5–8). A total of 20.8% (9/15) of the mutated lesions also had aneuploid cell populations. Table II shows a summary of lesions with *TP53* mutations, with DNA ploidy and mucosal morphology.

No correlation was detected between mutations in *TP53* and the accumulation of p53 in this material. Three progressor lesions had both a *TP53* mutation and accumulation of p53. All 3 lesions originated from separate colons and included two adenocarcinomas and one lesion indefinite for dysplasia. One of the adenocarcinomas was also aneuploid.

### Aurora A expression in UC progressors and non-progressors

We have previously demonstrated that the expression of Aurora A in UC mucosa is elevated compared to non-UC control samples (35). In the present study, Aurora A expression was not found to differ between the progressors and non-progressors, neither when including all types of progressor lesions, nor when only diploid, non-dysplastic progressor lesions were included.

**Figure 2:**
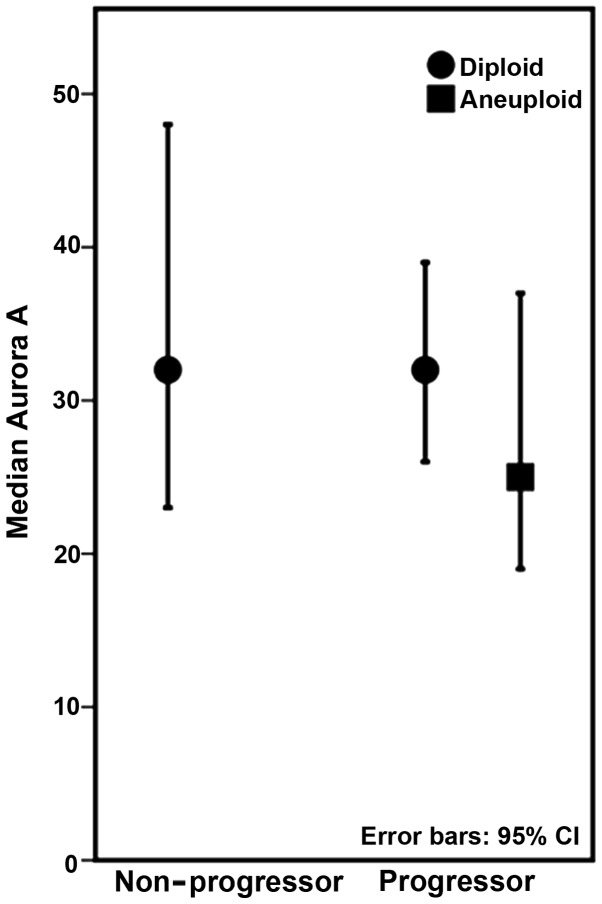
Aurora A expression and DNA ploidy status. Levels of Aurora A expression in ulcerative colitis non-progressors (all diploid) and in the diploid and aneuploid lesions from progressors. Within the progressors: lesions harbouring aneuploid populations had significantly decreased levels of Aurora A expression compared to diploid lesions (P=0.020). Median values with 95% confidence interval.

**Figure 3:**
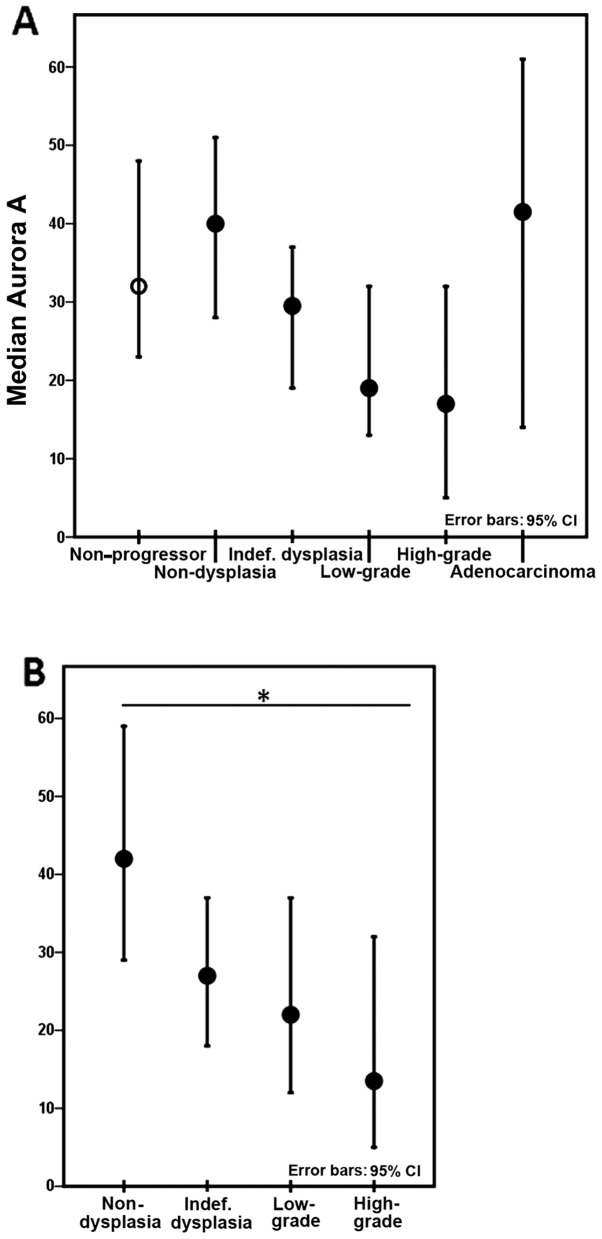
Aurora A expression in progressor ulcerative colitis (UC) colons with and without adenocarcinoma. Levels of Aurora A protein expression in lesions from (A) 10 non-progressor colons (white circle) and levels of Aurora A with increasing degrees of dysplasia within the 20 progressor colons, including those harbouring adenocarcinomas, and (B) the decreasing levels of Aurora A with increasing degrees of dysplasia in the 14 progressor colons remaining when excluding the colons harbouring adenocarcinomas (high- and low-grade dysplasia are combined in statistical analysis) (p=0.025). The asterisk (*) indicates statistical significance at a p-value of <0.05, and the horizontal bar indicates overall difference between groups [as detected by the linear mixed model (LMM) with Bonferroni post-hoc test]. Median values with 95% confidence interval are shown.

Within the progressors, we found a significant association between Aurora A and DNA ploidy status (p=0.020), with lower levels of Aurora A present in lesions harbouring aneuploid populations (Fig. 2). The expression of Aurora A within the progressor lesions decreased with increasing severity of dysplasia, but when accounting for patient variation this was not statistically significant. The lowest values of Aurora A expression were observed within high-grade dysplasia. Adenocarcinomas harboured increased levels of Aurora A expression (Fig. 3A). As only 6 lesions were diagnosed as high-grade dysplasia, these were combined with low-grade dysplasia for statistical purposes. Excluding the 6 colectomies harbouring adenocarcinomas, a significant decrease in Aurora A expression associated with increasing degrees of dysplasia was observed in the 14 remaining colectomies (p=0.025) (Fig. 3B).

### Expression of Aurora A associated with p53 accumulation/TP53 mutation

**Figure 4:**
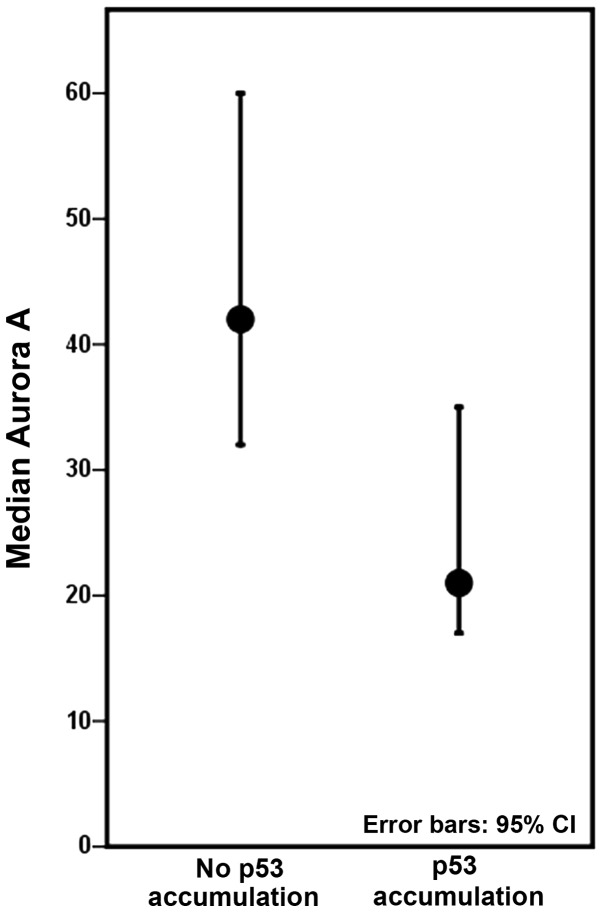
Aurora A expression and p53 accumulation. Aurora A expression in ulcerative colitis (UC)-progressor colons with lesions positive for p53 accumulation (>5% positivity) and in progressor colons without any lesions displaying p53 accumulation. Note the decreased expression of Aurora A in progressors without p53 accumulation, although not to a statistically significant degree when inter-patient differences were accounted for. Median values with 95% confidence interval are shown.

Colectomies harbouring at least one lesion with p53 accumulation displayed decreased levels of Aurora A, compared to colectomies with no p53 accumulation (Fig. 4), although not to a significant degree when inter-patient differences were accounted for. The expression of Aurora A was not significantly associated with the p53 mutation status in our study material.

## Discussion

It has long been known that protein levels of Aurora A are upregulated in the majority of solid tumours (31,44), linked to CIN and aneuploidy (33,45) and associated with a poor prognosis (46). It is also known that Aurora A is mapped to chromosome 20q13.2, a region highly amplified in, for example, sporadic colorectal cancers (32). UC colonic mucosa is subjected to rapid cell division (47) and high levels of oxidative stress (48), regardless of its status as progressor or non-progressor. Oxidative stress has been shown to induce spindle checkpoint override in cell lines, as it can inhibit the anaphase-promoting complex/cyclosome (APC/C) (49). Aurora A in normal functioning cells is targeted by APC/C for degradation during late mitosis, a function essential for mitotic exit. Persistent Aurora A may be able to prolong the anaphase and induce separation of chromatids (50). Both progressors and non-progressors in our material presented elevated levels of Aurora A compared to non-UC control samples, but only progressors revealed dysplastic development and DNA ploidy changes. This may suggest that the general increase in Aurora A levels observed in UC colonic mucosa is consistent with enhanced spindle checkpoint activity as a natural response to an accelerated cellular proliferation, as well as elevated levels of oxidative stress. Other factors however, are most likely also required to override the checkpoint function, inducing CIN and DNA aneuploidy.

We have previously presented findings of similar levels of human telomerase reverse transcriptase (hTERT) protein expression and equal shortening of mean telomere length in the colonic mucosa of progressors compared to non-progressors from the same UC patient material (36,37). These results are in accordance with UC being a disease that accelerates the ageing of the colonic mucosa (51); however, these parameters are unable to differentiate a progressor from a non-progressor UC colon. Likewise, our results indicate that the expression of Aurora A is not an ideal biomarker for differentiating progressor from non-progressor UC colons.

In this study*, TP53* mutations were detected in both progressors and non-progressors, consistent with the observation that the frequency of *TP53* mutation increases after at least 10 years of UC duration, and without association to malignancies (52), and the observation that *TP53* mutations are frequent in the inflamed tissue of UC colons (53). Of note, the majority of mutations were found within the non-progressors; however, no p53 accumulation was observed in the non-progressors. The reason for this is unclear. Lack of such correlation has been previously shown at the single crypt level in UC (54). The lack of detectable p53 may be due to nonsense mutations and premature stop codons, rather than a missense mutation; since with a missense mutation, the accumulation of p53 is to be expected. It has been observed that less than 20% of *TP53* mutations of human cancers are nonsense mutations or stop codon mutations (55,56). It has also been observed that the mutation of *TP53* occurs prior to loss of heterozygosity in the colonic mucosa of UC progressors (57), and our results may be indicative of an early mutation of a single *TP53* allele, whereas the remaining allele provides functional p53 in these non-progressors. Since we did not sequence the cases positive for *TP53* mutation, this aspect of our study remains unclear.

Pre-colectomy biopsies from the patients included in our study made it possible to track p53 positivity retrospectively. A total of 11 patients displayed p53 accumulation in the pre-colectomy biopsies. All 11 had developed progressor traits by colectomy. Six cases had indeed developed adenocarcinomas. The finding that only progressors showed p53 accumulation indicates that p53 accumulation may be a potential biomarker of a progressor colon, which is consistent with previous reports of p53 expression associating with dysplastic development in UC (58–60).

The expression levels of Aurora A within the progressors did not differ to a statistically significant degree when a comparison was made between the advancing degrees of dysplasia, including adenocarcinomas. However, when the 6 colectomy specimens with cancer were removed, a statistically significant decrease in Aurora A expression was observed with increasing levels of dysplasia. Adenocarcinomas presented elevated levels of Aurora A compared to dysplastic lesions (Fig. 3A). In addition, Aurora A expression was also significantly associated with DNA aneuploidy within the progressors (Fig. 2). This association was masked when progressors and non-progressors were combined (35). In our material, the aneuploid lesions had decreased levels of Aurora A compared to diploid lesions, again with the exception of adenocarcinomas, where the aneuploid cancers had elevated levels of Aurora A compared to the diploid cancers (data not shown). As colon cancer has been shown to harbour high levels of 20q amplification (32), a trait not often observed within the non-cancerous UC mucosa (2), this may be an explanation for the elevated levels of Aurora A in adenocarcinomas compared to the dysplastic lesions in our study material.

Recently, a study of Aurora A expression in dysplasia and cancer in gastric mucosa demonstrated increased levels of Aurora A in dysplastic gastric lesions (61). As this is in contrast to our findings in the UC mucosa, it may indicate that different mechanisms are involved in dysplastic development in the colonic and gastric mucosa.

p53 has been shown to be an important negative regulator of Aurora A. Loss of p53 may lead to the abnormal regulation of Aurora A and dysregulated mitosis (29,62,63). An increase in Aurora A expression may induce a protective mechanism, oncogene-induced senescence, against malignant development, possibly dependent on the loss of functional p53 (64–66). As our data show decreased Aurora A expression in areas harbouring aneuploid populations, it may be possible that the development of CIN and aneuploidy is necessary to overcome this protection. Our results showing that non-progressor lesions with wild-type *TP53* have higher Aurora A levels than mutated *TP53* non-progressor lesions, [although the difference was not statistically significant when inter-patient differences were re-accounted for (data not shown)], are also consistent with this hypothesis.

Wild-type p53 is difficult to detect in normal unstressed cells. The detection of p53 protein becomes possible due to the extended half-life of a mutated, non-functioning protein or by the stabilisation of p53 as a natural response to, for example, cellular stress and inflammation (30,67). p53 accumulation is also a known response to excess shortening of telomeres (68,69). As we have previously shown that the mucosa of UC progressor cases harbours significantly more ultra-short telomeres than those found in the non-progressor cases (36), this could indicates that telomeric repeat-induced activation of p53 is a possibility in our progressor cases. The lack of detectable p53 in non-progressors perhaps also indicates that the elevated levels of Aurora A in the non-progressors phosphorylate p53, targeting it for degradation. This is consistent with a previous report, demonstrating that Aurora A phosphorylates p53 at Ser315, leading to murine double minute 2 (MDM2)-mediated ubiquitination and degradation of p53 (62).

Our findings of no p53 accumulation detected in the non-progressors differ from those of previous studies (70). This may be due to our definition of a non-progressor; we included only non-dysplastic patients [described as having regenerative or inflamed mucosa by two experienced pathologists (O.P.C. and S.N.A.)] with no detectable DNA aneuploidy. We selected this definition as it has been shown that even UC patients with only one lesion indefinite for dysplasia, or with DNA aneuploidy alone, may develop adenocarcinoma (71,72).

In conclusion, our findings indicate that p53 accumulation may be a good biomarker for progressor UC cases, as no accumulation was detected in the non-progressors, and the progressors showed p53 accumulation in biopsies collected several years prior to colectomy. The expression of Aurora A did not differ between progressor and non-progressor UC colectomies. Within the progressor cases, the levels of Aurora A were decreased in association with both aneuploidy and dysplasia, but increased in adenocarcinomas. p53 and Aurora A appear to regulate each other in a different manner in progressors and non-progressors.

## Figures and Tables

**Table I:** Immunohistochemistry results from p53-positive lesions (n=27) in progressors.

	>5% p53 staining	
	
	Diploid	Aneuploid
Non-dysplasia	8	0
Indefinite dysplasia	5	1
Dysplasia	5	2
Adenocarcinoma	3	3

**Table II:** Ulcerative colitis colectomy lesions with mutated *TP53*.

		Mutated *TP53*	
		
Colon	Morphology	Diploid	Aneuploid
Non-progressors	Non-dysplasia	14	0
Progressors	Non-dysplasia	2	4
	Indefinite dysplasia	3	2
	Dysplasia	0	1
	Adenocarcinoma	1	2
